# Two new sponge species (Demospongiae: Chalinidae and Suberitidae) isolated from hyperarid mangroves of Qatar with notes on their potential antibacterial bioactivity

**DOI:** 10.1371/journal.pone.0232205

**Published:** 2020-05-13

**Authors:** Bruno Welter Giraldes, Claire Goodwin, Noora A. A. Al-Fardi, Amanda Engmann, Alexandra Leitão, Asma A. Ahmed, Kamelia O. Ahmed, Hadil A. Abdulkader, Halah A. Al-Korbi, Hala Sultan Saif Al Easa, Nahla O. Ahmed Eltai, Pejman Hanifi-Moghaddam

**Affiliations:** 1 Environmental Science Centre, Qatar University, Doha, Qatar; 2 Huntsman Marine Science Centre, St. Andrews, New Brunswick, Canada; 3 University of New Brunswick, Saint John, New Brunswick, Canada; 4 Biomedical Science Department, College of Health Science, Qatar University, Doha, Qatar; 5 Department of Chemistry and Earth Sciences, College of Arts and Sciences, Qatar University, Doha, Qatar; 6 Biomedical Research Centre, Qatar University (QA), Doha—Qatar; Universita degli Studi di Genova, ITALY

## Abstract

This study presents the taxonomic description of two new sponge species that are intimately associated with the hyperarid mangrove ecosystem of Qatar. The study includes a preliminary evaluation of the sponges’ potential bioactivity against pathogens. *Chalinula qatari* **sp. nov**. is a fragile thinly encrusting sponge with a vivid maroon colour in life, often with oscular chimneys and commonly recorded on pneumatophores in the intertidal and shallow subtidal zone. *Suberites luna* **sp. nov**. is a massive globular-lobate sponge with a greenish-black colour externally and a yellowish orange colour internally, recorded on pneumatophores in the shallow subtidal zone, with large specimens near the seagrass ecosystem that surrounds the mangrove. For both species, a drug extraction protocol and an antibacterial experiment was performed. The extract of *Suberites luna* **sp. nov.** was found to be bioactive against recognized pathogens such as *Staphylococcus epidermidis*, *Staphylococcus aureus* and *Enterococcus faecalis*, but no bioactive activity was recorded for *Chalinula qatari* **sp. nov.** This study highlights the importance of increasing bioprospecting effort in hyperarid conditions and the importance of combining bioprospecting with taxonomic studies for the identification of novel marine drugs.

## Introduction

The Persian-Arabian Gulf (PAG) is considered an extreme marine environment due to its hyperthermic and hypersaline conditions [1–3]. The environment in the southwestern coast of the PAG is particularly extreme. This shallow-water region and the associated mangrove settings has hyperarid conditions with temperature and salinity reaching values as high as 49°C and 75 ppt [4–7], levels much higher than the East coast of the Gulf [1–3,7–11]. The southwestern coast forms an isolated marine province with a high rate of marine endemism and lower species richness than the eastern coast of PAG, the latter receives an influx of waters from the Indian Ocean which results in a higher diversity of species [1,12–15]. The high rate of endemism found in the western coast of PAG, and the as yet, low number of taxonomic descriptions [1,14] for the region, indicate potential for the discovery of species new-to-science.

Marine ecosystems have considerable potential for bioprospecting, and several new drugs are described and isolated every year, yet these natural resources, which can produce economic and societal benefits, remain largely unexplored [16–20]. A significant majority of new marine natural products have come from sponges (Phylum Porifera) [21]. Chemical compounds isolated from sponges have been found to have anti-inflammatory, antibiotic, anticancer and anticoagulant properties [22–30]. Sponges are multicellular invertebrates [31–33] that have evolved as filter feeders in aquatic environments. Sponges naturally process a huge volume of water daily and as a consequence, may concentrate a wide variety of pathogens [34,35]. Due to this, sponges have developed effective defence systems based on bioactive secondary metabolites including antibacterial substances [33,36].

Despite their economic importance, virtually nothing is known about sponge diversity in the coastal areas in the Gulf, with only a few sponge records from the Arabian Sea and adjacent areas [1,28,37,38]. Environmental stress has been shown to concentrate toxins in sponges [39], and higher temperatures to be related with the bioactivity [21]. Therefore, the study of marine sponges in the extreme, hyperarid conditions found in the Southwest of PAG has potential for both the discovery of potential bioactive metabolites and species new to science.

The aims of this study are to describe two new sponge species and provide a preliminary evaluation of their bioactivities against pathogens.

## Material and methods

### Study area

**Fig 1:**
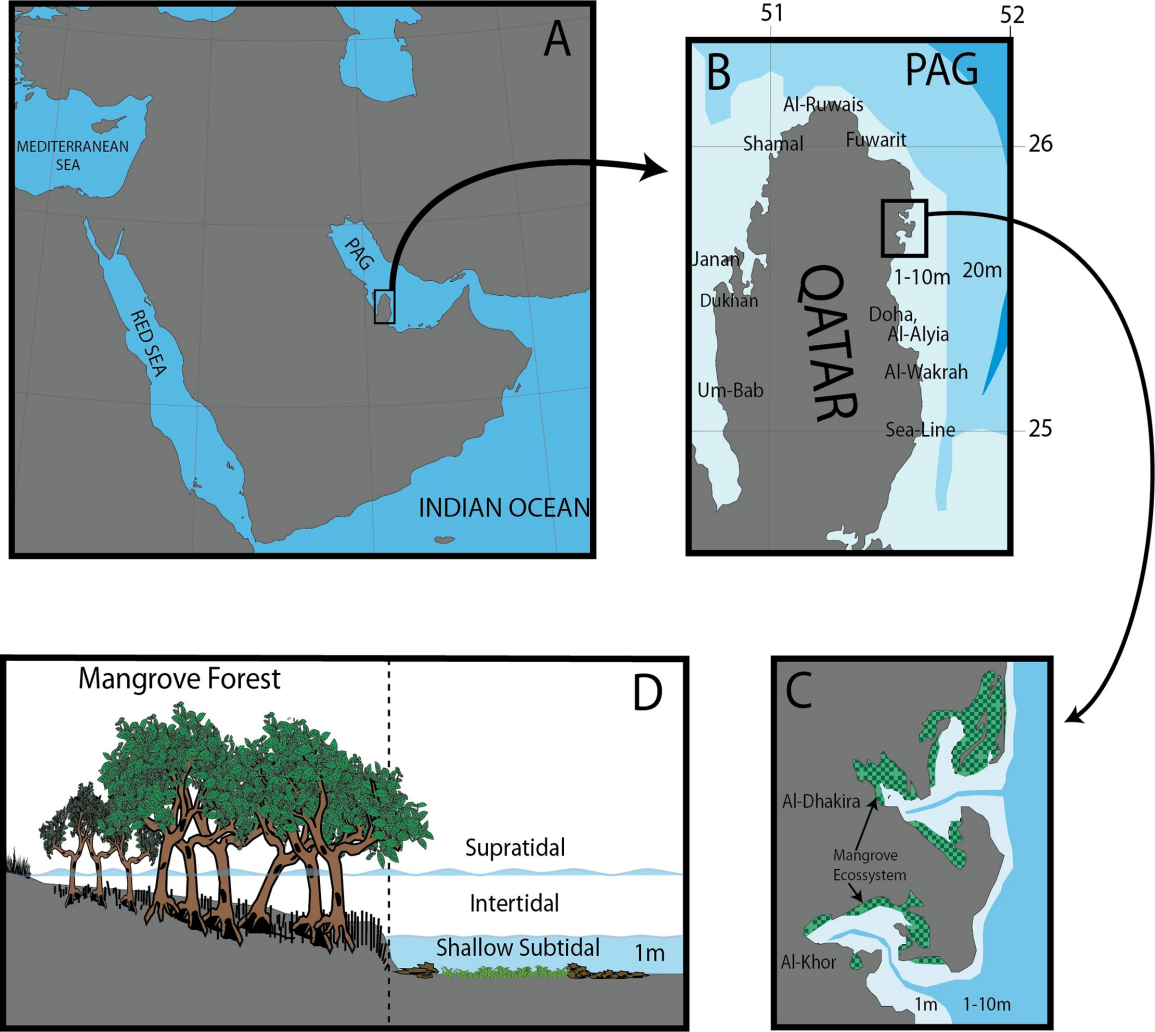
Collection localities in Qatar: (A) the location of Qatar within the Persian-Arabian Gulf (PAG); (B) the location of the studied mangrove settings and the other locations around Qatar that was searched for sponge species; (C) the studied mangrove settings in Al-Khor and Al-Dhakira highlighting the large area with shallow depth around the mangrove; (D) schematic profile of the mangrove ecosystem in the coastal intertidal zone with the forest area and the shallow subtidal zone with patches of seagrass and oyster-beds (rocks).

Shallow-water hyperarid mangrove ecosystems were studied at Al-Khor (25.69502778, 51.54694444) and Al-Dhakira (25.749228, 51.539267), Qatar. These areas do not experience any input of fresh water, but saline tidal channels are present. Areas of seagrass and oyster beds, interspersed with rocky substrate, surround and extend out from the mangroves in the shallow subtidal zone (<1 m) (Fig 1). The coastal zones of Qatar are characteristic by gently sloping shores and a large tidal range which result in large intertidal and shallow subtidal zones.

### Taxonomy and systematics

Sponges were collected in the intertidal and subtidal zones in the studied arid mangrove ecosystem. Most specimens were collected by snorkelling and freediving at the edge of tidal mangrove channels. Field studies did not involve endangered or protected species and there is no specific permission required for collection of Porifera in these locations. Specimens were photographed *in situ* using underwater cameras (Mark-ii and Fantasea housing FG7X-II). Large pieces of each species were transported to the laboratory and preserved in 70% ethanol. Methods for identification followed standard taxonomic procedures [31,32,40]. In the laboratory, thick longitudinal and cross sections were hand-cut using a scalpel, dehydrated in 98% alcohol, clarified in clove oil and mounted in Canada Balsam on microscope slides. These were used to examine the choanosomal and ectosomal skeleton. A small piece of tissue was dissolved in bleach to make a slide of the sponge spicules, the resulting spicules were washed in several changes of water and alcohol then mounted using Canada Balsam on microscope slides. The spicule and skeleton slides were observed and photographed using a compound microscope (Olympus CX22Led with an attached Nikon 7200 and an Olympus BX53 with camera DP73) and scanning electronic microscope (FEI Quanta-200). Spicule measurements were made using Olympus cellSens software and are presented as minimum length (mean length) maximum length by minimum width (mean width) maximum width, n = X. Specimen vouchers were deposited in the marine collection of the Environment Science Centre at Qatar University (ESC-QU). Holotypes of each new species have been donated to the Natural History Museum of London (NHMUK).

### Nomenclature acts

The electronic edition of this article conforms to the requirements of the amended International Code of Zoological Nomenclature, and hence the new names contained herein are available under that Code from the electronic edition of this article. This published work and the nomenclatural acts it contains have been registered in ZooBank, the online registration system for the ICZN. The ZooBank LSIDs (Life Science Identifiers) can be resolved and the associated information viewed through any standard web browser by appending the LSID to the prefix "http://zoobank.org/". The LSID for this publication is: urn:lsid:zoobank.org:pub:91890F03-5B54-4826-8280-C30E93E02405. The electronic edition of this work was published in the PlosOne journal with an eISSN 1932-6203 and has been archived and is available from the following digital repositories: PubMed Central, LOCKSS.

### Ecological description

After the first taxonomic identification, more than 50 freediving and snorkelling were performed in the tidal channels surrounding mangroves, and seagrasses, to identify zonation and distribution of the described species. In addition several dives were undertaken in shallow subtidal zones around Qatar, including Um-Bab, Dukhan and Janan Island in the west Coast, Shamal, Al-Ruwais and Fuwarit in the North coast and Al-Khor, Al-Dhakira, Doha, Al-Alyia Island, Al-Wakrah and Sea-Line in the east coast (Fig 1). Visual identification of sponges was performed based on the general shape, texture and colour of the described new species. Ecological information from these surveys is presented in the taxonomic description section.

### Antibacterial experiments

Details about the extraction methods of the chemicals from the studied sponges, the bacterial strains that were used (17 bacteria species), and the methodology used to identify the antibacterial bioactivity of the studied species are provided in the supporting information (S1 File). Methods and procedures based in references [23,26,41].

## Results

### Systematics

**Phylum Porifera Grant, 1836**

**Class Demospongiae Sollas, 1885**

**Subclass Heteroscleromorpha Cárdenas, Pérez & Boury-Esnault, 2012**

**Order Haplosclerida Topsent, 1928**

**Family Chalinidae Gray, 1867**

**Genus *Chalinula* Schmidt, 1868**

*Chalinula qatari* Giraldes & Goodwin 2020 **sp. nov.**

urn:lsid:zoobank.org:act:C22F9008-0031-4B10-9A6E-78498B8794A7

Fig 2

**Fig 2:**
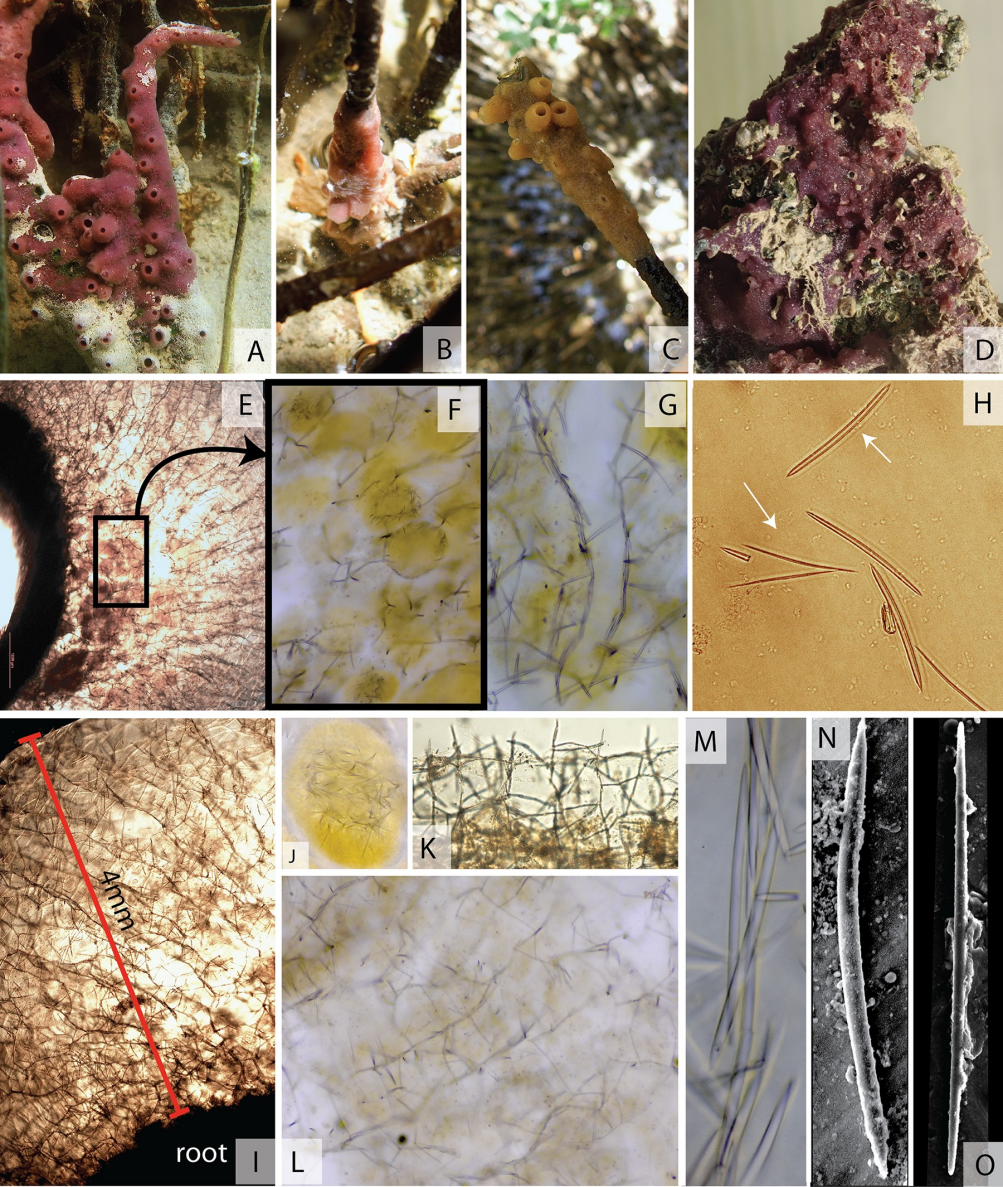
*Chalinula qatari* sp. nov., morphology, skeleton and spiculation. Morphology: Living specimens (A) attached to mangrove pneumatophores in the riparian zone, (B, C) in the intertidal zone, (D) under limestone in the channels between the mangroves. Skeleton and spicules: (E) choanosomal skeleton; (F) embryos; (G) ascending spicule tracts; (H) oxeas, showing immature thinner forms; (I) choanosomal skeleton showing thickness of encrustation on a mangrove root; (J) embryo; (K) Cross section of ectosome (specialised ectosomal skeleton absent); (L) choanosomal skeleton showing length of secondary spicule tracts; (M) close up of ascending primary spicule tract. Electronic microscopy of (N) large, thick oxea (O) thinner oxea.

#### Material examined

**Holotype.** NHMUK 2020.3.26.1 (ESC-QU00674) Al-Khor, Qatar, Arabian-Persian Gulf, 25.69502778, 51.54694444, 0.3 m, collected from pneumatophores in tidal-channels, May 2018. **Paratypes.** NHMUK 2020.3.26.2 (ESC-QU00420), Al-Dhakira, Qatar, Arabian-Persian Gulf, 25.749228, 51.539267, intertidal zone collected encrusting pneumatophores June 2015, 1 specimen; ESC-QU01327, Al-Dhakira, Qatar Arabian-Persian Gulf, 25.749228, 51.539267 (<1 m), collected encrusting pneumatophores, Feb 2019, 3 specimens.

#### Morphology

A thinly encrusting sponge with a thickness of around 4 mm (Fig 2I) and a maximum observed diameter of 40 cm. Oscular chimneys were present on some specimens. These had the form of small cones around 6 mm in diameter with an elevation of around 8 mm. Oscules were 2–5 mm in diameter. Oscular chimneys were observed mainly in the specimens in the mangrove roots (Fig 2A–2D).

#### Surface

Surface uneven.

#### Consistency

Compressible, very soft and fragile, easily damaged.

#### Colour

Most living specimens are a vivid maroon colour (Fig 2A, 2B and 2D). However, those living in stressful situations, such as intertidal specimens in summer conditions, may bleach to a pale yellow (Fig 2C). In alcohol specimens are pale yellow.

#### Skeleton

The choanosomal skeleton is an anisotropic reticulation with paucispicular primary tracts, 1–3 spicules in diameter (Fig 2G, 2L and 2M). The secondary tracts are unispicular (Fig 2L), usually about two spicules long (Fig 2L). There is no ectosomal skeleton (Fig 2K), the ends of the primary tracts of the choanosome project beyond the surface, rendering it slightly hispid.

#### Spicules

Oxeas, 69.2 (80.5) 96.2 μm length by 1.1 (2.5) 4.0 μm width, n = 25 (Fig 2H, 2M and 2N). Mature fusiform oxeas with 83 (86.4) 96.2 μm length by 3.0 (3.4) 4.0 μm width (Fig 2H and 2N); while young spicules commonly observed were shorter, thinner, and more sharply pointed with 69.2 (74.1) 77.8 μm length by 1.1 (1.4) 1.9 μm width (Fig 2H and 2O). No microscleres.

#### Ecology

Found growing on the pneumatophores of mangrove *Avicennia marina* (Forssk.) Vierh., in the intertidal and subtidal zones along tidal channels (Fig 2A–2C), and on the underside of limestone rocks in tidal channels (Fig 2D). Also found in seagrass and algal beds connected directly with the mangrove habitat.

#### Etymology

Named for the general type locality, Qatar, and the colouration, which is similar to that of the Qatari flag.

#### Distribution

Currently only known from the holotype and paratype localities in the mangroves at Al-Dhakira and Al-khor, planted mangrove in the Al-Wakrah in the south of Doha, and in the mangroves at Shamal in the north-east of Qatar. All locations are on the east coast of Qatar, south-western coast of the Arabian/Persian Gulf.

#### Antibacterial Bioactivity

Extracts from *Chalinula qatari* **sp. nov.** did not show any antibacterial bioactivity against the test pathogens.

#### Remarks

No significant differences in skeletal morphology or spiculation were observed between the paratypes. The proportion of smaller young oxeas did vary amongst the paratypes; with each specimens presenting a different ratio of large and thin spicules. Embryos with young spicules were visible in some individuals (Fig 2F and 2J) these were always concentrated in the basal layer (Fig 2E).

The possession of an isodictyal skeleton of diactinal megascleres, and a regular anisotropic reticulation with recognisable ascending primary tracts, places this species in Order Haplosclerida Topsent, 1928, Sub-order Haplosclerina Topsent, 1928. The presence of a choanosomal skeleton with unispicular secondary lines assigns this species to Family Chalinidae Gray, 1867. Within the Chalinidae we assign this species to genus *Chalinula* on the basis that the secondary tracts of the choanosomal skeleton are mostly two spicules long and multispicular fibre tracts are not present throughout the sponge [42].

*Chalinula* has 25 currently accepted species worldwide [42], none of which have been recorded in the PAG. Three species occur in related biogeographic areas: *Chalinula camerata* (Ridley, 1884) from the Indian Ocean and Red Sea, *Chalinula confusa* (Dendy, 1922) from the Seychelles, and *Chalinula saudiensis* Vacelet, Al Sofyani, Al Lihaibi & Kornprobst, 2001 from the Red Sea [43–45]. In addition to the *Chalinula* species, since the taxonomy of this family is confused, we considered species from closely related genera. *Haliclona (Reniera) debilis* Pulitzer-Finali, 1993, which has similar colour and also occurs in mangroves, is known from the north-west Indian Ocean [46,47]. A comparison of these four species with the new *C*. *qatari* **sp. nov.** is presented in Table 1. The main characteristics that differentiate *Chalinula qatari* **sp. nov.** from those congeners are the colour in life (maroon), the presence and sizes of the oscular chimneys, the encrusting thickness, the size of the spicules (oxeas) and the habitat preferences, dwelling in the intertidal and shallow subtidal zones at hypersaline mangroves.

**Table 1:** Taxonomic comparisons of the new species *Chalinula qatari* sp. nov. with target congener from family Chalinidae.

	*Chalinula qatari* sp. nov.	*C*. *saudiensis*	*C*. *camerata*	*C*. *confusa*	*Haliclona (Reniera) debilis*
		Vacelet et al., 2001	(Ridley, 1884)	(Dendy, 1922)	Pulitzer-Finali, 1993
**ECOLOGY**					
**ecosystem**	Mangrove	Black Coral Reef			Seagrass (Halimeda) adjacent to mangroves
**Depth/zone**	Mid to low Intertidal/shallow subtidal	20–30 m			Upper eulittoral
**substratum**	Living substrate (mangrove roots); and limestones	dead coral heads			Muddy sand
**MORPHOLOGY**				
**Colour**	Maroon,	Blue	Pale brown–soft leather	Dark brown	Bright pinkish purple
**Thickness (mm)**	encrusting (4)	encrusting (10–20)	Subcylindrical Lamellae (1–2)	Erect, branched	Coalescing tubes
**Projections**	Oscular chimneys sometimes present (8 mm high; 6 mm width).	none		Longitudinal series of vents	Coalescing tubes 30 mm high; 5mm wide
**Oscules (mm) diameter**	2–5	2–6			
**SKELETON**					
**Choanosomal Primary Tracs**	anisotropic reticulation; paucispicular	Reticulation; paucispicular	Polispicular	paucispicular	
**Secondary tracts**	unispicular generally two spicules long	unispicular generally two spicules long	multispicular	unispicular	
**Ectosomal skeleton**	absent	absent	Present	absent	
**SPICULES**					
**Type**	oxeas	oxeas	oxeas	oxeas	
**Length (μm)**	83(86.4)96.2 mature; 69.2(74.1)77.8 young	110–181 mature; 60 young	180	150 mature	70–85
**Width**	3.0(3.4)4.0 mature; 1.1(1.4)1.9 young	1.5–4.5 mm mature; 0.5young	7	6 mature	3–4.5

**Order Suberitida Chombard & Boury-Esnault, 1999**

**Family Suberitidae Schmidt, 1870**

**Genus Suberites Nardo, 1833**

*Suberites luna* Giraldes & Goodwin 2020 **sp. nov.**

urn:lsid:zoobank.org:act:174C5AD0-3132-4D07-A172-2014A77CBDC8

Fig 3

**Fig 3:**
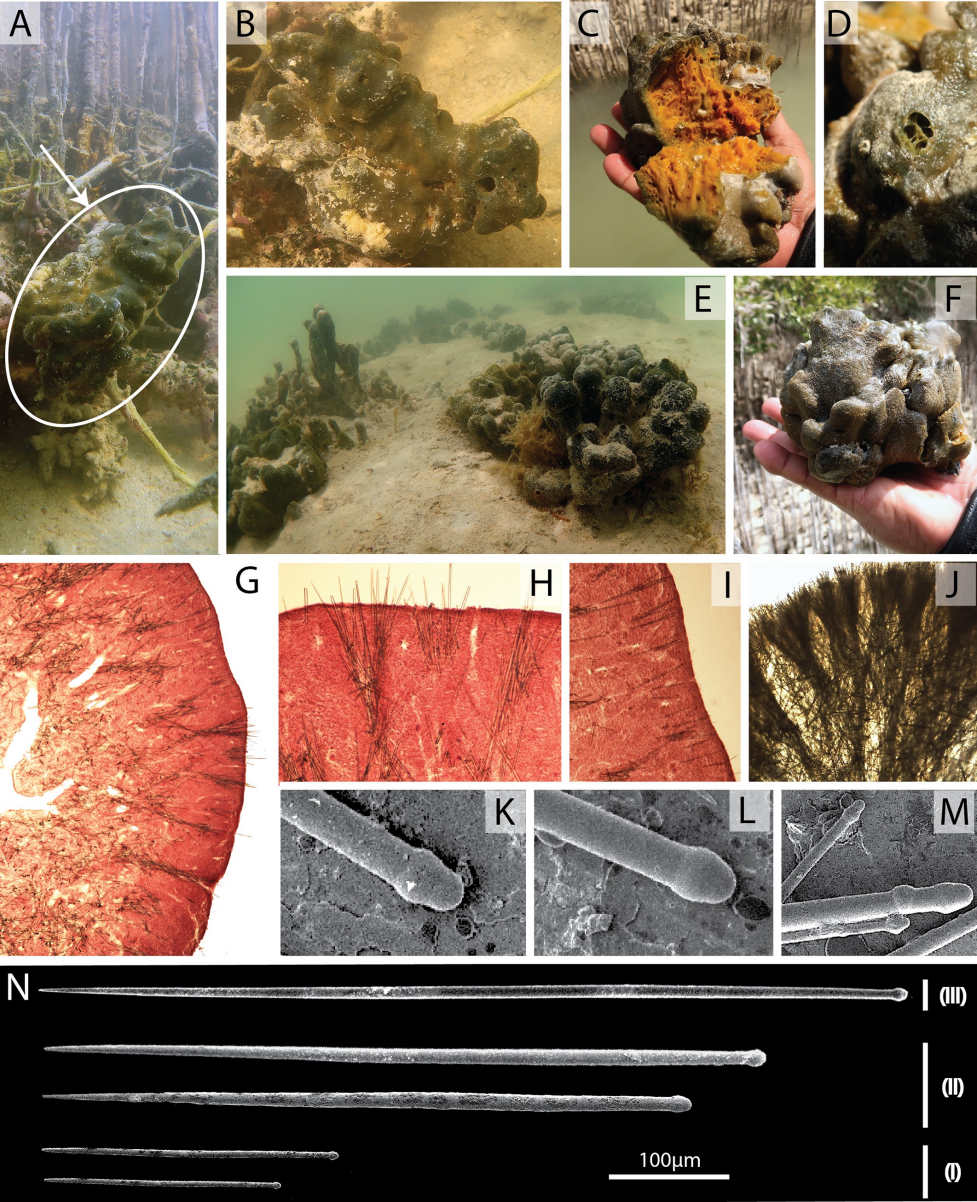
*Suberites luna* **sp. nov.**, morphology, skeleton and spiculation: (A, B) growing on mangrove pneumatophores in the riparian zone; (C) just collected and cut; (D) large compound oscule; (E) large specimens close to seagrass; (F) specimen just collected. Slides of fresh specimens, (G) cross section of choanosomal skeleton; (H) plumose choanosomal skeleton in cross section, (I) palisade of subtylostyles in the ectosome. Slide in cross section of dried specimen showing plumose choanosomal skeleton (J). Electronic Microscopy, (K, L, M) showing different head shapes of the subtylostyles; (N) the subtylostyles types (I), (II) and (III).

#### Material examined

**Holotype.** NHMUK 2020.3.26.3 (ESC-QU0067) Al-Khor, Qatar, Arabian-Persian Gulf, 25.69502778, 51.54694444, <1 m collected from pneumatophores in tidal channels bordering hyperarid mangroves, May 2018; **Paratypes.** NHMUK 2020.3.26.4 (ESC-QU 00419) Al-Khor, Qatar, Arabian-Persian Gulf, 25.749228, 51.539267, 1.5 m, collected from rock/sand substrate in the hyperarid mangrove bay, June.2015, 1 specimen; ESC-QU 01432, ESC-QU 01436 and ESC-QU 01437, Al-Dhakira Qatar, Arabian-Persian Gulf, 25.749228, 51.539267, 1–2 m, collected from shells, soft rock on sand substrate in the seagrass peripheral to the hyperarid mangrove, May 2018, 3 specimens.

#### Morphology

Massive globular-lobate sponge (Fig 3A–3F), with some large specimens 20–60 cm diameter and 10–20 cm high. The sponge exterior is dense and compact. The interior choanosomal tissue has many pores and is cavernous. Oscules are infrequent, the largest observed was around 8 mm in diameter (Fig 3D) and was on the apex of a lobe.

#### Surface

Velvety surface with macroscopically smooth appearance (Fig 3A–3F).

#### Consistency

Compact, firm, slightly compressible and elastic; hard to tear. A slime is produced when torn.

#### Colour

Live colour is greenish-black and internally a yellowish orange (Fig 3A–3F). When preserved in alcohol the tissue becomes grey.

#### Skeleton

Plumose skeleton with ascending tracts of large subtylostyles, 10 to 50 spicules wide (Fig 3J and 3H). Ectosomal skeleton formed of a palisade of smaller subtylostyles (Fig 3I).

#### Spiculation

**Fig 4:**
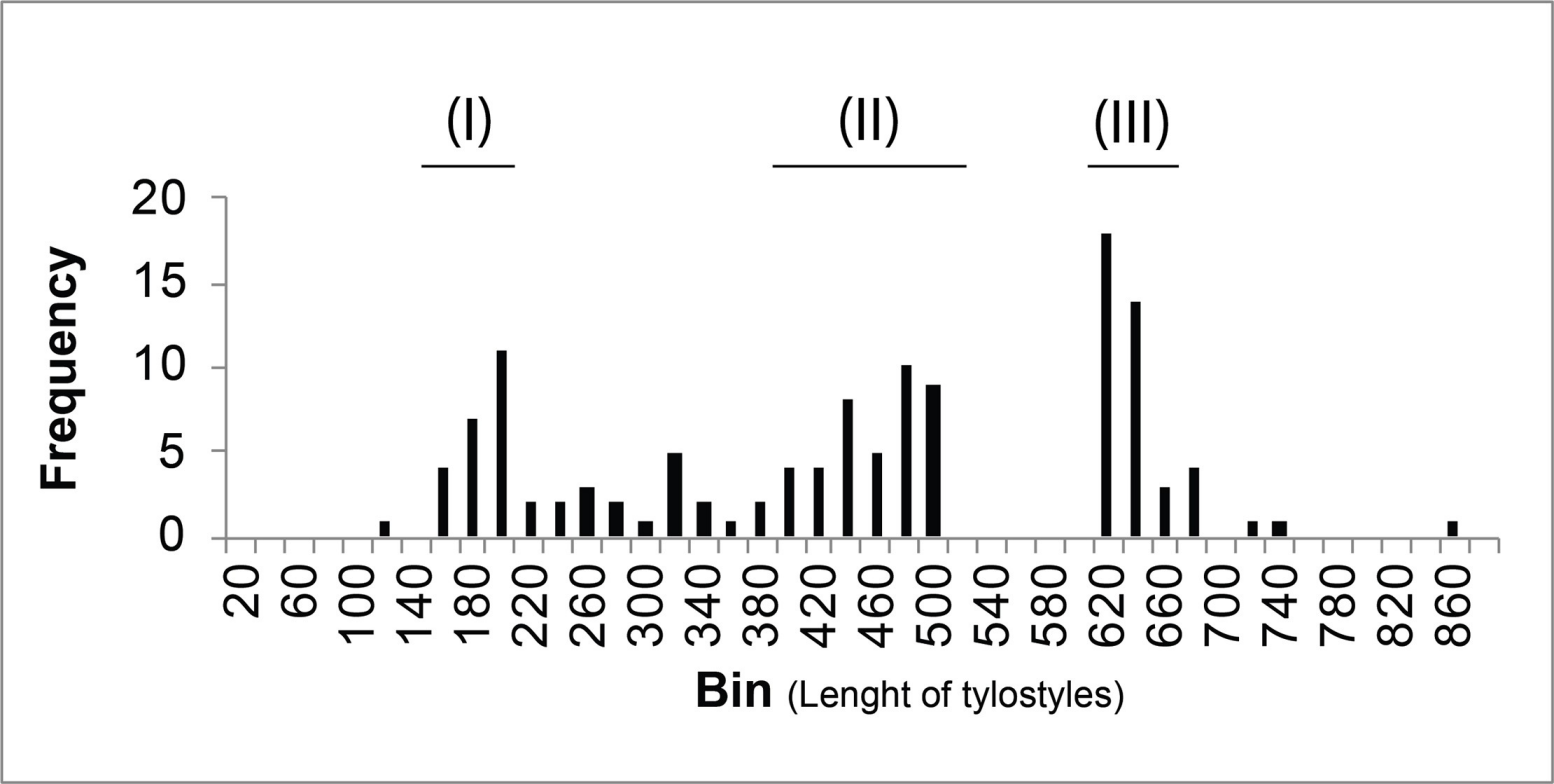
Histogram of subtylostyle length (n = 234, showing three potential size categories. I-III.

Subtylostyles, 10 (491) 843 μm by 2.9 (6.4) 13.1 μm (n = 234) (Fig 3N). A multimodal pattern of spicule length was observed (Fig 4), with three main sizes of tylostyles (subtylostyles): (I) smaller spicules with 110 (174) 196 by 2.9 (4.2) 5.9 μm, most likely ectosomal in distribution; (II) robust subtylostyles with 400–500 by 5 (7.8)13.1 μm width, found in the sub-ectosomal choanosomal skeleton; (III) long subtylostyles, >600 μm length by 5.6 (6.6)10.4 μm (Fig 3N), part of the deep choanosomal skeleton forming the ascending tracts in the plumose skeleton.

#### Ecology

Found on hard substrates in mangrove and seagrass habitats in the subtidal zone. Observed on the pneumatophores of *Avicennia marina* in the channels of the riparian zone of the arid mangrove ecosystems (Fig 3A). Several times this species was found close to *Chalinula qatari* **sp. nov.** Very abundant with large specimens (more than 50 cm diameter) in the subtidal zone around the mangroves (Fig 3E) and at the edges of the seagrass habitat. Found in soft sediment, but mostly attached to small pieces of hard substrate within the sediment, such as small soft-rocks and shells. There was a higher abundance of this species at sites with low current.

#### Etymology

This species was nicknamed the ‘moon-surface sponge’ by the collectors due to its appearance. The name reflects both this and the importance of the moon in the Muslim culture.

#### Distribution

Recorded from mangrove ecosystems on the east coast of Qatar from Shamal to Al-Wakrah, south-western coast of the Arabian/Persian Gulf.

#### Antibacterial bioactivity

**Fig 5:**
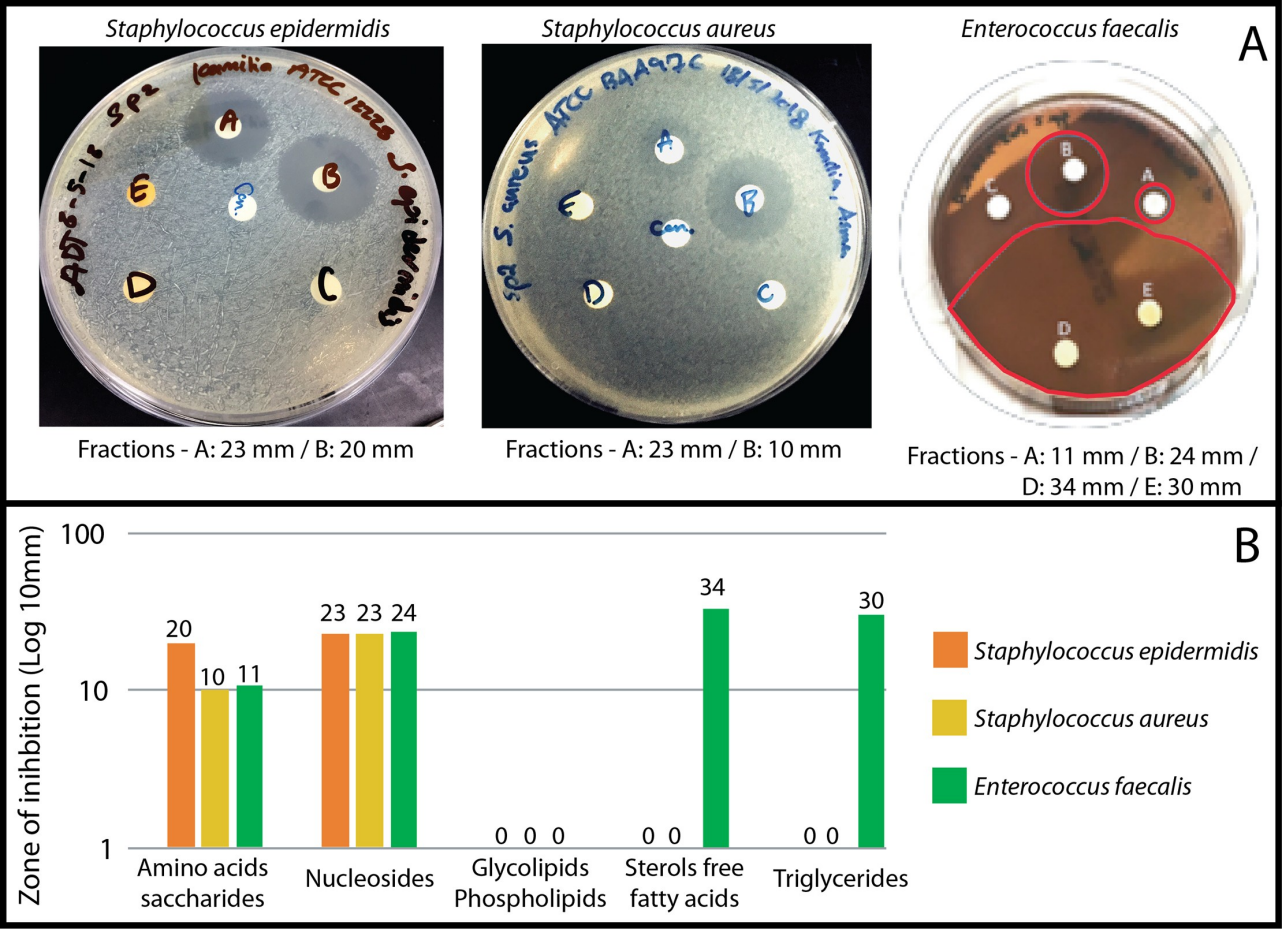
Bioactivity experiment with *Suberites luna* sp. nov.. (A) zone of inhibition (highlighted in red) over three species of bacteria; (F) and the chart highlighting the zone of inhibition.

Fractions A and B of *Suberites luna* **sp. nov**. showed antibiotic activity against three species of bacteria (*Staphylococcus epidermidis*, *Staphylococcus aureus*, *Enterococcus faecalis*), 17% of those tested (Fig 5). Fractions D and E (see S1 File) were effective against only 6% (*Enterococcus faecalis*) of the bacteria tested in this study (Fig 5A and 5B). Fraction C (S1 File) showed no antibacterial activity against any of the bacterial strains.

#### Remarks

Significant differences in skeleton and spiculation of the paratypes was not observed. However, there was some variation in external form with some specimens being much larger and more lobate than others (Fig 3E). This species is included within the Family Suberitidae Schmidt, 1870 and Genus *Suberites* Nardo, 1833 due to its massively globular-lobate shape, possession of a spicule complement consisting only of tylostyles, and the presence of an ectosomal palisade formed of bouquets of smaller tylostyles than those of the choanosome [48]. Genus *Suberites* has 80 species worldwide [48–50] but only a few congeners have been previously recorded in the Indian Ocean and Red Sea [37,50–53]. These are *S*. *bengalensis* Lévi, 1964 recorded from India (1190 m depth) (see [49]; *S*. *clavatus* Keller, 1891 and *S*. *tylobtusus* Lévi, 1958 from the Red Sea; *S*. *radiatus* Kieschnick, 1896 from Indonesia [50]; and *S*. *diversicolor* Becking & Lim, 2009 from Singapore, Indonesia, Vietnam, Australia [49] and more recently recorded from the East of PEG [54]. A sixth species *Suberites carnosus* (Johnston, 1842) was previously recorded from the Indian Ocean, more specifically from the Seychelles and Minicoy Islands and the coast of India (Mumbai) [55–57]. However, *S*. *carnosus* and all the variations within this species complex including *var*. *depressus*, *var*. *incrustans*, *var*. *novaezealandiae* and *var*. *ramosus* are not now considered to inhabit any marine province in the Indian Ocean [50] and therefore this species and its variants were discarded from this comparison. A comparison with the aforementioned biogeographically related species is presented in Table 2. Based in the spicules size and types, the main divergent characteristic that differentiate those species and one of the only descriptions recorded for all congeners.

**Table 2:** Taxonomic comparisons of the new species *Suberites luna* sp. nov with target congener from family Suberitidae.

	*Suberites luna* sp. nov.	*S*. *bengalensis* Lévi, 1964;	*S*. *diversicolor* Becking & Lim 2009:855	*S*. *clavatus* Keller, 1891	*S*. *tylobtusus* Lévi, 1958; Samaai et al 2017
**SPICULES**					
**Megascleres**	Tylostyles/Subtylostyles (3 sizes)	Subtylostyles (2 sizes)	Tylostyles	Tylostyles	Tylostyles (2sizes) and tylostrongyles
**Length (**μm**)**	110(491)843 Small: 110–220 Medium: 400–500 Large: 600–843	Small: 280–1000 Large: 1200–1600	165-499-810	300-449-530	Small: 350–450 Large: 440–556
**Width (**μm**)**	2.9(6.4) 13.1 Small:2.9–5.9 Medium: 5–13.1 Large: 5.6–10.4	Small: 7–20 Large:30–32	2.5–8.9–17.5	5–9.8–15	Small:10–11 Large: 15

We could not compare *Suberites radiatus* because, as noted by Becking and Sim [49], the original description is extremely brief and vague and the type specimen seems to have been lost. Some biogeographically related species mentioned in the Table 2 also differ from *Suberites luna* **sp. nov.** in terms of morphology and habitat preferences. *Suberites bengalensis* is a deep-sea species recorded from 1190 m [49]; *S*. *tylobtusus*, when living, is a bright orange colour [50]. *Suberites diversicolor* has spicules of a similar size range to *S*. *luna*. However, it has a more uniform distribution of spicules across this size range whereas there is a gap in size of around 200 μm between the smallest category of (ectosomal) spicules of our specimens and those found in the larger two categories (choanosomal). *Suberites diversicolor* is also much more variable in colour, ranging from purple-brown and olive green to red-orange, where our specimens are always greenish-black. In addition, the holotype locality of *S*. *diversicolor* is an anchialine lake, the other areas it has been recorded from (Indonesian coastal mangroves, Singapore, lake systems in Vietnam, and a man-made pool in Darwin northern Australia) also had low salinities, and it seems to be restricted to areas with salinities between 26 and 29 psu [49]. *Suberites luna* **sp. nov.** was recorded from shallow coastal waters with salinities from 42 to >60 psu and water temperatures reaching 36°C. Studies have demonstrated that different sponge species inhabit waters of differing salinities [58] and lethal effects was recorded when exposing some sponge species to elevated temperatures [59]. We argue that *S*. *diversicolor* would be unlikely to be found in the conditions found in the PAG. Although *S*. *diversicolor* was reported from the PAG from Bushehr, Iran [54], we believe these records need to be revisited. It has been noted that even in the type locality *S*. *diversicolor* may represent a species complex [60].

Summarizing, the main characteristics that differentiate *Suberites luna* **sp. nov.** from the related congeners are the internal (yellow) and external (dark olive) colour in life, the massive globular-lobate shape, the spicule size range and number of spicule categories (Table 2) and the habitat preference, dwelling in the subtidal zone in a hypersaline mangrove ecosystem.

## Discussion

The discovery of *Suberites luna* **sp. nov.** and *Chalinula qatari* **sp. nov.** on mangroves on the west coast of the PAG highlights the lack of taxonomic study of sponge species in the Gulf but also the biogeographic isolation of the studied hyperarid mangrove habitats. These two species new to science, together with the other endemic species that have been found in this habitat [15] support the concept that the west coast of PAG is an isolated marine province. Theoretically, the intense hyperarid conditions found in the west coast of PAG create a biogeographic barrier that isolates an endemic biodiversity adapted to the intense temperature and salinity conditions [1,3]. The deeper waters and constant water input from the Indian Ocean result in less extreme arid conditions on the eastern coast of the PAG, and this area shares several species with tropical Indian Ocean areas (e.g. gastropods and decapods) [12,13]. The high temperatures and salinities found on the western PAG coast might kill non adapted sponge species, as was demonstrated for tropical sponge species reaching 33°C [59], preventing colonisation by sponges from neighbouring provinces. Recent studies on the biodiversity of bioturbating crabs [61], based in the same arid mangrove setting, support the theory that the southwest coast of PAG is an isolated marine province. A mangrove setting in an isolated marine province that houses an abundant endemic shrimp *Palaemon khori* De Grave & Al-Maslamani, 2006 [7,15] that occurs only in this mangrove setting in Qatar and remains absent in the entire Arabian Gulf [62] (BWG pers. Observ.). It is possible the two new sponge species are also endemic to this mangrove setting in the type locality. If they are it would bring the number of endemic species known to three. This highlights the conservation importance of this forest ecosystem in a desert region. Further study of the western PAG sponge fauna is needed to fully understand its biodiversity and biogeographic affinities with neighbouring regions.

*Suberites luna* **sp. nov.** exhibited antibacterial activity against three common pathogenic gram-positive bacterial species, *Staphylococcus aureus*, *S*. *epidermidis* and *Enterococcus faecalis*. Although this is a preliminary study it highlights the potential of the toxins produced by *Suberites luna* **sp. nov.** for the development of a new antibacterial drug, including drugs for resistant bacteria. Future studies are required to chemically isolate the toxin of *Suberites luna* **sp. nov.** and evaluate its uses in treatment of bacteraemia and other bacterial infections. Despite the negative antibiotic effect of *Chalinula qatari* **sp. nov.** the fact other studies on the family Chalinidae have found metabolites indicate that it might merit future research. The sulphated sterol *Chalinulasterol*, has been isolated from the family Chalinidae [63]. Additionally, a unidentified species from family Chalinidae recorded in the PAG presented antifungal and antibacterial activity [64]. These results highlight the importance of increasing the effort in taxonomic study and study of the metabolites of the marine species of the west coast of the PAG.

## Supporting information

S1 File Antibacterial studies on extracts from *Chalinula qatari* sp. nov. and *Suberites luna* sp. nov. (DOCX)
