# Supplementary material for: Two new sponge species (Demospongiae: Chalinidae and Suberitidae) isolated from hyperarid mangroves of Qatar with notes on their potential antibacterial bioactivity
Source: PLoS One. 2020 May 13;15(5):e0232205. doi: 10.1371/journal.pone.0232205 (PMC7219822; doi:10.1371/journal.pone.0232205)
Supplement: S1 File — (DOCX) [file pone.0232205.s001.docx]

**Supporting Information – 1: Antibacterial studies on extracts from** *Chalinula qatari* **sp. nov.** and *Suberites luna* **sp. nov.**

**Material and Methods**

**Extraction for antibacterial Study**

Fresh material of each species was washed with autoclaved sea water, from the study area and kept at -80° C. Exactly 50 grams of frozen *Chalinula qatari* **sp. nov.** and *Suberites luna* **sp. nov.** were weighed and used for the extraction. The extraction process was performed according to [1][2] and yields of 2.109 mg and 2.189 mg dry extract were obtained for *Chalinula qatari* **sp. nov.** and *Suberites luna* **sp. nov.,** respectively.

The aqueous suspensions were loaded onto pre-packed CHROMABOND® HR-X cartridges (6 mL/500 mg) and submitted to a five-step elution protocol. After a preliminary desalting step with 2 mL of distilled water, fractionation of the organic components was achieved by elution with 100% H_2_O (fraction A, 18 mL) followed by solvent mixtures of increasing chromatographic strength from CH_3_OH/H_2_O (fraction B, 50:50, 24 mL) to CH_3_CN/H_2_O (fraction C, 70:30, 18 mL), 100% CH_3_CN (fraction D, 18 mL) and, finally, CH_2_Cl_2_/CH_3_OH (fraction E, 90:10, 18 mL). The polarity sequence of these eluents gave good recovery with a fair distribution of the major components in the five fractions.

**Bacterial Strains**

The 18 bacterial strains used in this research were obtained from American Type Culture Collection (ATCC) in form of dried loops and inoculated according to ATCC instructions (Table 1).

Table 1: List of bacterial strains used in the antibacterial experiment.

| **Bacterial Strain** | **ATCC number** |
| --- | --- |
| *Staphylococcus aureus* | ATCC BAA976 |
| *Listeria monocytogenes* | ATCC 7644 |
| *Staphylococcus epidermidis* | ATCC 12228 |
| *Enterococcus faecalis* | ATCC 29212 |
| *Streptococcus agalactiae* | ATCC 12386 |
| *Streptococcus pneumoniae* | ATCC 6303 |
| *Streptococcus pyogenes* | ATCC 19615 |
| *Lactobacillus plantarum* | ATCC 8014 |
| *Klebsiella pneumoniae* | ATCC 13883 |
| *Bacillus cereus* | ATCC 11778 |
| *Escherichia coli* | ATCC 11775 |
| *Proteus mirabilis Hauser* | ATCC 12453 |
| *Proteus vulgaris* | ATCC 6380 |
| *Salmonella enterica* | ATCC 13076 |
| *Shigella sonnei* | ATCC 25931 |
| *Staphylococcus epidermidis* | ATCC 12228 |
| *Pseudomonas aeruginosa* | ATCC 1744 |
| *Candida albicans* | ATCC 10231 |

**Antibacterial Experiments**

Using agar disc diffusion assay, each disk was impregnated with 250 μg sponge extract/fraction dissolved in diethyl ether as solvent, and allowed to air dry. Control disks contained diethyl ether only. The loop containing original ATCC strain was inoculated in 5 ml of nutrient broth and incubated in a shaker incubator at 37^o^C /150 RPM, overnight. The following day the culture density was adjusted to achieve an inoculum equivalent to 0.5 McFarland turbidity as compared to Wickerham Card. The bacterial plates were prepared by plating 20 μl of the bacterial suspension, 0.5 McFarland turbidity, on an appropriate agar plate [3]. The impregnated and control disks were placed on the freshly streaked plates using sterile forceps and incubated for 18–24 h at 37^o^C. The following day the zone of inhibition was measured and used to determine the relative antibacterial activity for each fraction against each strain [3]. Organic components in the sponge extracts were selectively eluted into the following five fractions: A) saccharides and amino acids, B) nucleosides, C) polar lipids, D) free fatty acids and E) triglycerides and other neutral lipids.

**Result**

The summary of the antibacterial experiment is presented in figure 5. Out of 17 strains exposed to different fractions isolated from the extract of two newly identified sponge species only *Suberites luna* **nov.sp** presented an active antibiotic effect over three different bacterial species. A well-defined zone of inhibition was recorded, in four fractions of the extract of *Suberites luna* **nov.sp.**which exhibited antibacterial activity to various degree against three gram-positive bacterial strains: *Staphylococcus epidermidis, Staphylococcus aureus, Enterococcus faecalis.* On the other side, the extract of *Chalinula qatari***nov.sp.** showed no antibacterial activity against the tested strains. The zone inhibition is summarized in figure 5 in the manuscript.

**References**

1. Shaari K, Kee CL, Rashid ZM, Tan PJ, Abas F, Raof SM, et al. Cytotoxic aaptamines from Malaysian Aaptos aaptos. Mar Drugs. 2009;7: 1–8. doi:10.3390/md7010001

2. Cutignano A, Nuzzo G, Ianora A, Luongo E, Romano G, Gallo C, et al. Development and application of a novel SPE-method for bioassay-guided fractionation of marine extracts. Mar Drugs. 2015;13: 5736–5749. doi:10.3390/md13095736

3. Beesoo R, Bhagooli R, Neergheen-Bhujun VS, Li WW, Kagansky A, Bahorun T. Antibacterial and antibiotic potentiating activities of tropical marine sponge extracts. Comp Biochem Physiol Part - C. Elsevier; 2017;196: 81–90. doi:10.1016/j.cbpc.2017.04.001
